# Efficacy of ruthenium coordination complex–based Rutherrin in a preclinical rat glioblastoma model

**DOI:** 10.1093/noajnl/vdz006

**Published:** 2019-05-28

**Authors:** Manjunatha Akathatti Munegowda, Carl Fisher, Daniel Molehuis, Warren Foltz, Mark Roufaiel, Jay Bassan, Mark Nitz, Arkady Mandel, Lothar Lilge

**Affiliations:** 1 Theralase Technologies Inc., Toronto, Ontario, Canada; 2 Princess Margaret Cancer Centre, University Health Network, Toronto, Ontario, Canada; 3 Department of Medical Biophysics, University of Toronto, Toronto, Ontario, Canada; 4 Techna Institute, University Health Network, Toronto, Ontario, Canada; 5 Department of Radiation Oncology, University of Toronto, Toronto, Ontario, Canada; 6 Department of Chemistry, University of Toronto, Toronto, Ontario, Canada

**Keywords:** aminolevulinic acid, glioblastoma, orthotopic, photodynamic therapy, ruthenium

## Abstract

**Background:**

Glioblastoma is an aggressive brain cancer in adults with a grave prognosis, aggressive radio and chemotherapy provide only a 15 months median survival.

**Methods:**

We evaluated the tolerability and efficacy of the Ruthenium-based photosensitizer TLD-1433 with apo-Transferrin (Rutherrin) in the rat glioma 2 (RG-2) model. The specific tumor uptake ratio and photodynamic therapy (PDT) threshold of the rat glioblastoma and normal brain were determined, survival and CD8^+^T-cell infiltration post-therapy were analyzed. Results were compared with those obtained for 5-aminolevulinic acid (ALA)-induced Protoporphyrin IX (PpIX)-mediated photodynamic therapy in the same animal model. As both photosensitizers have different photophysical properties, the number of absorbed photons required to achieve an equal cell kill was determined for in vitro and in vivo studies.

**Results:**

A significantly lower absorbed energy was sufficient to achieve LD_50_ with Rutherrin versus PpIX-mediated PDT. Rutherrin provides a higher specific uptake ratio (SUR) >20 in tumors versus normal brain, whereas the SUR for ALA-induced PpIX was 10.6. To evaluate the short-term tissue response in vivo, enhanced T2-weighted magnetic resonance imaging (MRI) provided the spatial extent of edema, post PpIX-PDT at twice the cross-section versus Rutherrin-PDT suggesting reduced nonspecific damage, typically associated with a secondary wave of neuronal damage. Following a single therapy, a significant survival increase was observed in rats bearing glioma for PDT mediated by Rutherrin versus PpIX for the selected treatment conditions. Rutherrin-PDT also demonstrated an increased CD8+T-cell infiltration in the tumors.

**Conclusion:**

Rutherrin-PDT was well tolerated providing a safe and effective treatment of RG-2 glioma.

**Key points**

1. The selectivity of Rutherrin for the tumour is very high.
2. There is a significant improvement in the post-PDT survival.
3. Post-PDT the tissue inflammation is reduced compared to ALA-induced PDT.

**Importance of the Study**

The investigational photosensitizer Rutherrin is one of two photosensitizers able to cross the blood–brain barrier. Despite its size of ~1 kD, it is actively transported by transferrin. Additionally, through the high transferrin receptor expression, a favorable tumor-specific uptake is feasible. Compared to published PDT-glioma models in mice or rats using a single light treatment, animal survival was significantly longer versus controls and Aminolevulinic acid-induced protoporphyrin IX-mediated PDT. With these positive results in a murine model and Rutherrin’s ability to absorb light at longer wavelengths, its minimal skin sensitivity is seen in an unpublished clinical study, Rutherrin has high potential as a photosensitizer to target brain cancer.

Glioblastoma (GBM) is a highly aggressive and common type of brain cancer in adults with a grave prognosis.^1^ Historically with maximal safe resection surgery followed by aggressive radio and chemotherapy, the current median survival is only 15–18 months according to various studies.^2^ The majority of GBM patients are not curable with surgery alone as the tumor cells typically invade the surrounding brain and the contralateral side, moving along the white matter tracks, rendering complete resection difficult and generally non-attainable due to the need to preserve eloquent or vital volumes of the brain. Hence, radiotherapy and concomitant chemotherapy, adjuvant chemotherapy^3^; image-guided surgical resection,^4,5^ and intensity-modulated ionizing radiation therapy^6^ are generally used as additional primary therapies.^7^ Chemotherapies efficacy is limited as the CNS is an immune privileged site with the blood–brain barrier (BBB) regulating the trafficking of molecules to and from the brain, limiting the size and charge of therapeutic molecules reaching the brain parenchyma.^8,9^ Novel therapeutic strategies are warranted to target GBM and overcome these obstacles. Photodynamic therapy (PDT),^10^ Immunotherapy,^11^ BBB disrupting technologies using focused ultrasound^12,13^ are being evaluated to treat GBM.

PDT is an approved treatment for some cancer indications, including GBM in Japan. PDT is based on the combination of a photosensitive drug and light photons to generate cytotoxicity inside cells. PDT induces destruction of tumor tissue by three main pathways, reviewed in Allison and Moghissi^14^: (1) By the production of reactive oxygen species which directly cause the death of tumor cells via apoptosis or necrosis. (2) In the case of vascular acting photosensitizers; photoactivation causes bleeding and thrombosis of blood vessels, leading to the death of cancer cells through deprivation of nutrients to the cells. (3) PDT can activate an immune response against tumor cells through acute inflammation mediated release of damage-associated molecular patterns (DAMPs) and cytokine release, resulting in an influx of leukocytes which contribute to tumor destruction and stimulation of the immune system to recognize and eliminate cancer cells as reviewed by Allison and Moghissi.^14^

Here we report on the performance of TLD1433, a ruthenium transition metal-based coordination complex as photosensitizer^15^ in the rat glioma-2 (RG-2) model. TLD1433 is currently in clinical studies for nonmuscle invasive bladder cancer. The TLD1433 chemical formula is [Ru(4,4′-dimethyl-2,2′-bipyridine)^2^(2-(2′,2′′:5′′,2′′′-terthiophene)-imidazo[4,5-f])] Cl_2_, its active center comes from the metal to ligand transfer of the photon’s quantum energy. It was shown that an improved absorption coefficient is obtained following complex formation with transferrin. Thus, TLD1433 is the active compound in the drug now referred to as Rutherrin.^16^ Rutherrin uptake into tumor cells is first either by; passive diffusion into the brain parenchyma through the leaky BBB, or across the BBB through the active uptake of transferrin. Beyond the leaky BBB, Rutherrin is actively endocytosed via the tumor cell’s transferrin receptor (TfR) enabling uptake by microinvasion distal from the tumor core.^16^

The use of 5-aminolevulinic acid (ALA), a pro-agent metabolized by most cells into Protoporphyrin IX (PpIX) can as act as a fluorescence contrast agent or as the photosensitizer and has been evaluated clinically for glioma PDT.^17^ In 2017, ALA-induced PpXI was approved by the US Food and Drug Administration as an adjunct for the visualization of malignant tissue during surgery in glioma patients.^18^ Uptake of ALA into GBMs can likewise occur via the intact BBB. Through the use of ALA in image-guided resections most neurosurgeons are familiar with ALA-induced PpIX and its selectivity to malignant tissues.^19^

Here, we are using a single 5-ALA-induced PpIX-mediated PDT as a comparator to evaluate the utility of Rutherrin-mediated PDT to treat preclinical gliomas.

The photosensitizers have different photophysical and photochemical properties. ALA-induced PpIX absorbs light strongly at the soret band (405 nm) and the Q-Bands, at 505, 540, 580, and 630 nm.^20^ In contrast, Rutherrin absorbs light throughout the visible spectral range and also demonstrates a sufficient absorption coefficient at NIR wavelengths (808 nm) permitting the treatment of larger tissue volumes in vivo, compared to 630 nm^21^ light (Supplementary Table 1). Direct comparison of the two photosensitizers’ efficacy is difficult due to the different tissue concentrations and molar extinction coefficients. Hence, we used the photodynamic threshold model as a basis for comparison.^22^

RG-2 tumor model was used to evaluate Rutherrin’s efficacy and cytotoxicity in vitro and for orthotopic tumors gown in the immune competent Fisher rats. In vivo treatment response was evaluated using MRI, histology and survival post-PDT. Efficacy is expressed using the photodynamic threshold model^22,23^ and compared to the efficacy of ALA-induced PpIX.

## Materials and Methods

### Cell Culture

The RG-2 cell line (RG-2) using passages 19–30, was grown and maintained in DMEM supplemented with 10% fetal bovine serum, 2-mM glutamine, and penicillin/streptomycin (all from Life Technologies). New cell stock was used when morphological changes in their growth were observed.

### Photosensitizers

GMP grade TLD1433 synthesized by Sigma Aldrich Fine Chemicals was provided by Theralase Inc.. For in vitro studies, TLD1433 was diluted to 2 mM in autoclaved water and further diluted in media as required. In vivo formulation with human apo-transferrin (Sigma-Aldrich) was prepared as described previously.^16^ ALA was purchased from Sigma-Aldrich.

### In vitro PDT

RG-2 cells were plated at 10 000 cells per well in 96 well plates. For PDT, cells were incubated with Rutherrin at varying concentrations 0–500 nM in phenol-red free DMEM media with 10% fetal bovine serum, 2 mM glutamine and penicillin/streptomycin (all from Life Technologies). For in vitro ALA-induced PpIX-mediated PDT, cells were incubated with ALA (Sigma-Aldrich) at concentrations from 0–6000 μM. Each plate contained a solvent (ddH_2_O) and cell death control (4% methanol). Cells were incubated with Rutherrin or ALA for 4 hours and rinsed to remove the unbound drug. PDT was performed using custom-built irradiators containing either one 530 nm or one 635 nm emitting LED per well (both from Newark Corp) providing an irradiance of 360 mW cm^−2^ for Rutherrin or 75mW cm^−2^ for PpIX-mediated PDT, requiring either 56 seconds to deliver 20 J cm^−2^ equivalent to a photons density of 5.334*10^19^ hv cm^−2^ for Rutherrin or 133 seconds to deliver 10 J cm^−2^ at a photons density of 3.19 *10^19^ hv cm^−2^ for PpIX. The respective PDT-dose is obtained by multiplying the photon density with the respective molar extinction coefficient for Rutherrin 530 nm (8427.4 M^−1^ cm^−1^) or PpIX (5121 M^−1^ cm^−1^). Cell viability was measured 24 hours post light exposure by Presto Blue metabolic assay (Invitrogen Corp.),^24^ using a Flexstation 3 plate reader (Molecular Devices) at ten reads per well. The cells killed percentage was plotted on a logarithmic scale as a function of photosensitizer absorbed photons. GraphPad Prism Software (Version 6.0 Mac; GraphPad) was used for sigmoidal regression analysis to determine the in vitro LD_50_ dose of both photosensitizers.

### In Vivo Orthotopic RG-2 Tumor Model Induction and Monitoring

In vivo procedures were approved by the University Health Network Animal Ethics committee certified by the Canadian Council on Animal Care to follow ethical requirements for animal welfare in medical research. Orthotopic RG-2 tumors were generated as described before^10^ by injection of 5000 cells in sterile DPBS via a burr hole 1 mm into the neocortex of CDF Fischer rats (Charles River Laboratories), using a stereotactic frame. The burr hole was closed with bone wax and the skin sutured. Tumor growth was monitored weekly starting at day seven post induction, using MRI until tumors reached 3-mm diameter to commence PDT. We had a 100% tumor induction success rate.

### PS Tissue Uptake Studies

RG-2 tumor bearing rats were injected IV with either 5 or 10 mg TLD1433/kg body weight (bw) equivalent Rutherrin. Rats were killed at either 4, 24, or 48 hours post-Rutherrin injection. The skull was opened, and tissue samples from RG-2 tumor, the contralateral frontal cortex and the cerebellum were harvested. The fresh tissue samples were weighed and digested with 2 mL of trace metal grade nitric acid (Sigma-Aldrich). Inductively coupled plasma mass spectrometry quantification of the ^101^Ru isotope followed established standard operating procedures and the measured ppb was converted using ppb approximate μg/L units and the dilution with 2 mL acid digest volume. The TLD1433 per sample [μg] = Ruthenium mass [μg] × 1007MW_TLD1433_[g/mole]/AM_Ru_[g/mole]. A minimum of n = 3 animals were used to determine the specific uptake ratio (SUR) given by the ratio of RG-2 target TLD1433 versus contralateral brain or cerebellum using average tissues concentrations. PpIX tissue concentrations were obtained by tissue solubilization and fluorescence quantifications as previously reported.^25^

### In Vivo PDT

PDT was performed when the orthotopic tumors reached 3 mm in diameter. For Rutherrin based PDT, an IV injection of Rutherrin formulation at 5 or 10 mg TLD1433/kg bw was given either 4 or 24 hours prior to light treatment. Animals were irradiated with 200 mW of 808 nm for 50 minutes delivering 600 J via an isotropic emitter (ISP0.85, Medlight) inserted in the superior portion of a tumor. For ALA-based PDT, an IP injection of ALA (pH 6.8, 62.5 mg kg^−1^) was given 4 hours prior to PpIX photoactivation with 635 nm at 18 mW for 22 minutes 13 seconds for 24 J. Following PDT, animals received analgesic 2 mg kg^−1^ dexamethasone daily for 3 days.^10^ The number of animals used for each group is indicated in Figure 6.

### MRI Scanning and Analysis

T2w and Gd-enhanced T1w MRI were performed as explained in the Supplementary Information. MRI tumor volume assessment was performed on days −4, −1, 10, and weekly after that. The geometric features of both acquisitions were matched (25.6 × 25.6 mm field-of-view, 128 × 128 matrix, 0.2 × 0.2 mm in-plane resolution, at least eighteen 0.5-mm thick slices were collected).

To assess intratumoral edema/nonspecific inflammation, ROIs were drawn around regions of visible hyperenhancement on day 1 and day 3 post-PDT on T2-weighted images, using MIPAV software (version 7.2.0 CIT-NIH). Unpaired *t* test was performed on the resulting ROIs volumes.

### Estimating the Absorbed Photon Density at the RG2 Tumor Boundary Using FullMonte Simulations

To compare the in vivo efficacy of TLD1433 with ALA-PpIX-mediated PDT, imaging-based light dosimetry simulations were conducted using the acquired MRI volumes described earlier and preprocessed by resampling of the MRI volumes to create isotropic voxel dimensions [0.2 × 0.2 × 0.2 mm], which were skull-stripped, and bias field corrected.^26,27^ The data were converted into an in silico model using a chain of open-source software (MATLAB, Convert3D (www.itksnap.org), itk-SNAP (www.itksnap.org), Logismos, ANTs (http://stnava.github.io/ANTs/), CGAL (www.cgal.org) Monte Carlo simulations of the Boltzmann (radiative transport) equation used FullMonte (www.gitlab.com/FullMonte/FullMonteSW), to determine the photon density at the boundary of necrosis. Calculations are based on a Tcl script (www.tcl.tk) our open-source kernel FullMonte takes the tetrahedral mesh, optical properties assigned to each discretized tisues for both treatment condition,^28–30^ and the light diffuser geometry as input parameters, reporting the photon packet counts, which traces the spatial photon density distribution.

When combined with postprocessing steps, the probably absorbed photon distribution is calculated as a dose volume histogram and can be visualized as an unstructured grid using ParaView. The threshold values are computed based on the simulated photon density at the minimum and a maximum distance of the MRI enhancement zone using the tissue concentrations as determined earlier.

### Immunohistochemistry

For immunohistochemistry, formalin-fixed tissues were processed and paraffin-embedded, 4-μm thick sections prepared on charged slides and dewaxed by five changes of xylene, hydrated through decreasing grades of alcohols in water. Cleared sections were blocked with 3% hydrogen peroxide to remove endogenous peroxidase. Antigen retrieval was achieved with Tris-EDTA pH of 9.0.

CD71 (transferrin receptor) staining: Primary CD71 antibody (ThermoFisher, cat no. 13–6800 clone H68.4) was added at 1:300 for 1 hour. HRP-Anti-mouse IgG was used as the secondary antibody. Color development was done using DAB (DAKO, cat no. K3468)

CD3 and CD8 dual staining were performed as per MACH 2 Doublestain Cocktail kit (Intermedico cat no. BC-MRCT525G). A cocktail of both primary antibodies, CD8a (Biolegend, 1 in 100 cat no. 201701, Clone OX-8) and CD3 (1 in 300 DAKO, cat no. A0452) were incubated for 1-hour, and the MACH 2 Doublestain Cocktail (Intermedico cat no. BC-MRCT525G) added as per kit instructions. Colors development was achieved using DAB (DAKO cat no. K3468) for CD3and Warp Red (Intermedico cat no. BC-WR806H) for CD8, counterstaining with Mayer’s Hematoxylin. Sections were mounted with MM 24 Leica mounting medium (cat no. 3801120). CD3 and CD8 double-stained cells are CD8+ T-cells. Quantification was by the automated HALO software (Indica Labs).

### Statistical Analysis

Determination of the LD_50_ concentrations in vitro was based on a non-linear regression analysis performed using GraphPad Prism Software (Version 6.0 Mac, GraphPad). The in vivo Kaplan–Meyer survival curves were compared using the Mantel–Cox Log-Rank test.

## Results

### Rutherrin-PDT Requires a Lower Number of Absorbed Photons to Induce Cell Kill In Vitro

**Fig. 1:**
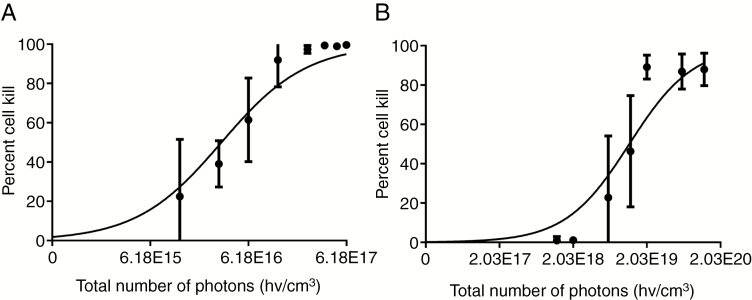
In vitro presto-blue cell kill assay comparing Rutherrin versus ALA-mediated PDT response. (A) The total number of photons required to achieve LD_50_ was 2.388*10^16^ hvcm^-3^ for Rutherrin. (B) LD_50_ was 1.539*10^19^ hvcm^-3^ for ALA.

Plotting cell kill data as a function of absorbed photons, using 8427.4 M^−1^ cm^−1^ and 5121 M^−1^ cm^−1^ (https://omlc.org/spectra/PhotochemCAD/data/149-abs.txt) as molar extinction coefficients for Rutherrin at 530 nm and PpIX at 630 nm, respectively, including considering the need of 8 ALA per PpIX^48^, resulted in the Rutherrin-PDT LD_50_ = 2.39*10^16^ hv cm^−3^ being three orders of magnitude lower than for ALA-PDT LD_50_ = 1.54*10^19^ hv cm^−3^ when assuming a complete conversion of the available ALA into PpIX (Figure 1).

### RG-2 Tumors Have a Very High Level of Transferrin ReceptorExpression

**Fig. 2:**
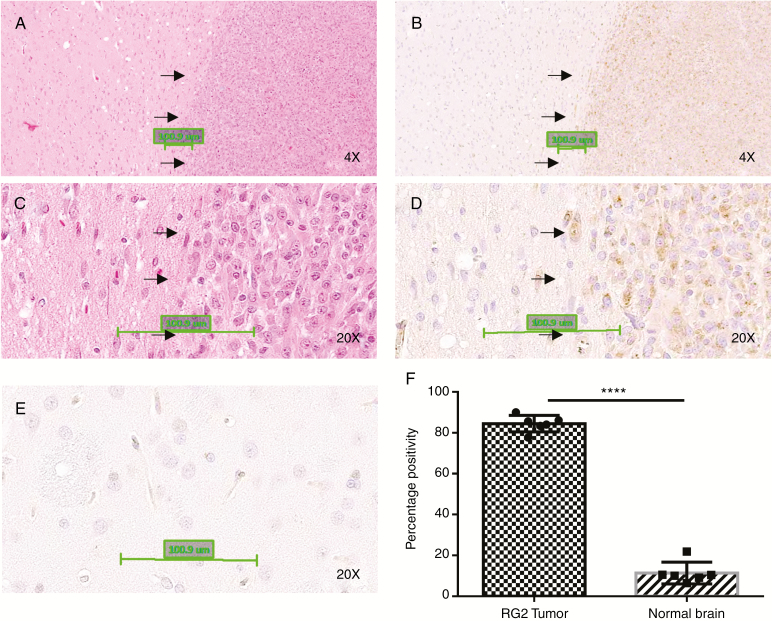
RG-2 tumors have significantly higher expression of the transferrin receptor. (A) and (C) are hematoxylin and eosin stained sections of a representative brain section with an RG-2 brain tumor; tumor border is marked with arrows. (B) and (D) are corresponding sections of (A) and (C), stained by immunohistochemistry for the transferrin receptor. Brown 3,3′-Diaminobenzidine (DAB) stained cells are positive for the transferrin receptor. (E) is showing a section from contralateral brain showing DAB negative cells. (F) depicts quantification of transferrin receptor positivity analyzed using Aperio image scope software on whole mount images.

TLD1433 was shown associate with Tf, could be internalized by cells via the TfR.^16^ CD71 stained immunohistochemistry sections from RG-2 carrying rats were scanned and CD71 positive pixel quantified. RG-2 tumors had a significantly higher TfR expression with 84% ± 4.03% positive pixels compared to the contralateral normal brain with less than 12% ± 5.32% positive pixels (*N* = 6) (Figure 2). Some high TfR expressing cells were noted in the normal brain adjacent to the tumors. These may be RG-2 cells invading normal brain (Figure 2D). We did not observe individual cells with high TfR staining in the contralateral brain (Figure 2E).

### Rutherrin Shows a High SUR in RG-2 Tumors

**Fig. 3:**
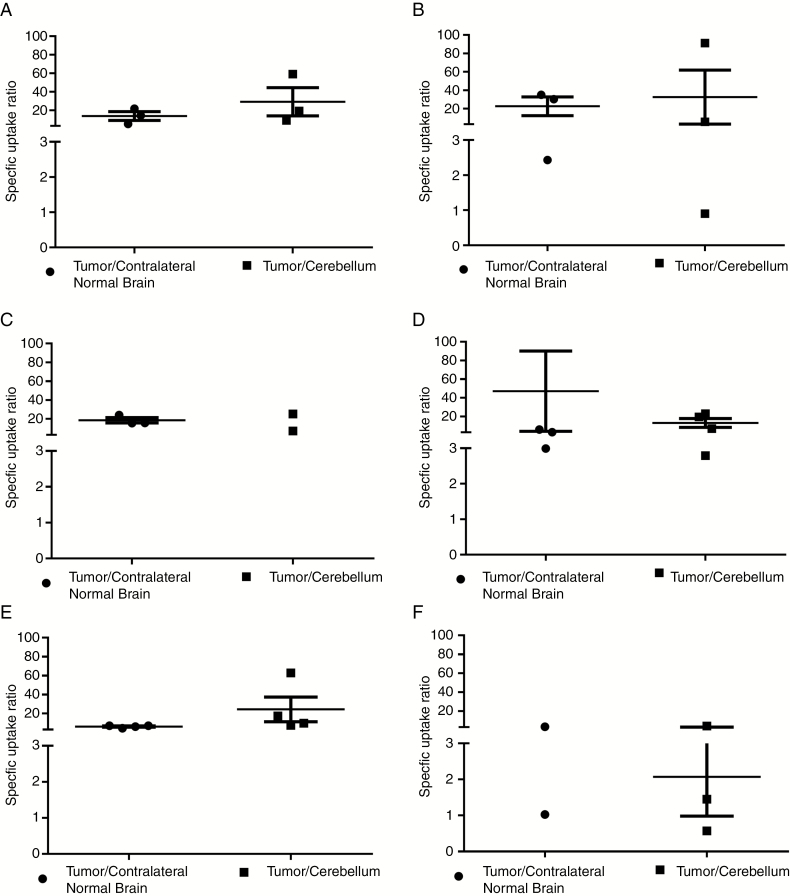
SURs are showing selective Rutherrin uptake by RG-2 tumors compared to contralateral normal brain area and cerebellum. Panels (A) and (B) are samples collected 4 hours post-Rutherrin injection, injected with 5 and 10 mg kg^−1^ bw TLD1433 in Rutherrin formulation, respectively. Panels (C) and (D) are samples collected 24 hours post-Rutherrin injection, injected with 5 and 10 mg kg^-1^ bw TLD1433 in Rutherrin formulation, respectively. Panels (E) and (F) are samples collected 48 hours post-Rutherrin injection, injected with 5 and 10 mg kg^−1^ bw TLD1433 in Rutherrin formulation, respectively.

The TLD1433 RG-2 over normal contralateral brain or cerebellum SUR was approximately 20 at 4 and 24 hours (Figure 3). One rat receiving 5 mg Kg^−1^ bw TLD1433 did not show uptake into the cerebellum at 24 hours (Figure 3C and Supplementary Table 2), preventing determination of the SUR variability for that group. At 48 hours post-Rutherrin injection, the SUR decreased but remained above 10, demonstrating a prolonged selectivity of TLD1433 toward RG-2 tumors (Figure 3). Only one rat receiving 10 mg Kg^−1^ bw of TLD1433 in the contralateral normal brain at 48 hours (Figure 3F and Supplementary Table 2) was available. The near to complete absence of TLD1433 in normal tissue indicates a very high selectivity toward the tumor. The SUR of TLD1433 is higher at all time points than those reported previously for PpIX following ALA administration in the same model.^10^

### Estimating the Absorbed Photon Density at the RG-2 Tumor Boundary Using FullMonte Simulations

**Fig. 4:**
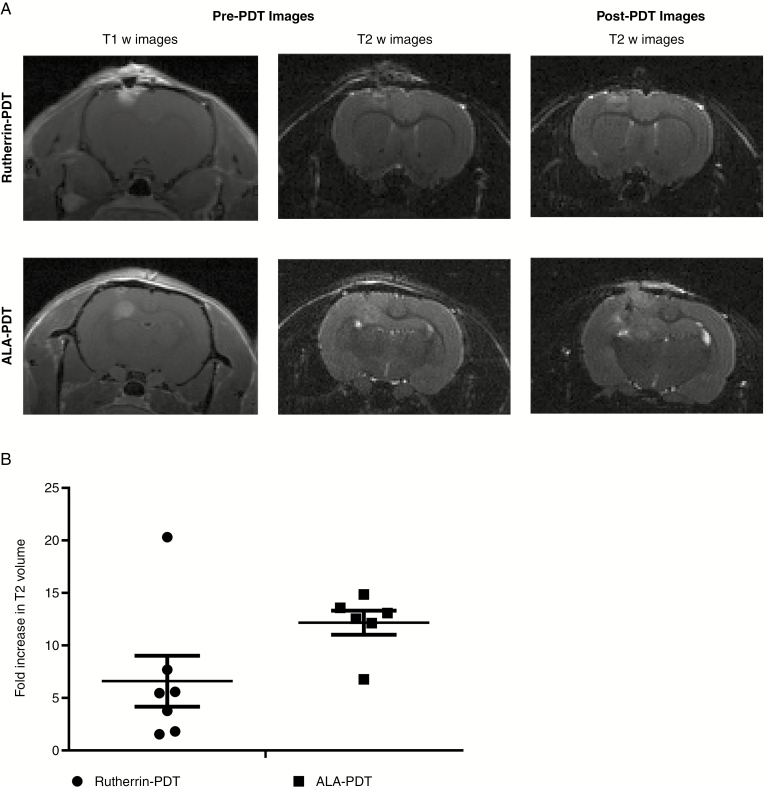
Rutherrin-mediated PDT induces less edema/inflammation following PDT treatment compared to ALA-PpIX-mediated PDT. (A) T1w and T2w images of a representative animal for Rutherrin and ALA-PpIX PDT, showing both pre-PDT and 3-day post-PDT images. (B) Fold change in mean T2 enhancement volume for both the treatments.

Using pre-PDT MRI images in silico 3D models were generated,^26^ and the photon density throughout the tumors and normal area of the brain was light simulated by FullMonte, using tissue optical properties from literature,^28–30^ with the absorption coefficient corrected based on the PS tissue concentrations obtained here, see Supplementary Table 3 or as published for ALA.^10^ The placement of the light source was approximated as used in the in vivo studies see Figure 4. Simulations indicate that for 808 nm activated Rutherrin-PDT the absorbed photon concentration could be over one order of magnitude higher than for ALA-PDT, for an equal photon number launched (Supplementary Figure 1), due to the lower effective attenuation coefficient for 808 versus 635 nm, see Supplementary Table 4.

### Rutherrin-Mediated PDT Resulted in a Confined T2-Weighted Enhancement at 72 hours Post Light Exposure

T2-weighted image enhancement is associated with increased free water content and commonly associated with an injury inflicted edematous brain, termed as the area of secondary brain injury.^31^ Edema is also a contributor to nonspecific damage throughout the brain due to increased intracranial pressure. Quantitative T2 volume changes between the pre- and post-PDT MRI images show that the T2 volume increased significantly more following PpIX-PDT compared to Rutherrin-PDT (Figure 4).

### Rutherrin-PDT Resulted in a High Infiltration of CD8+ T-Cells

**Fig. 5:**
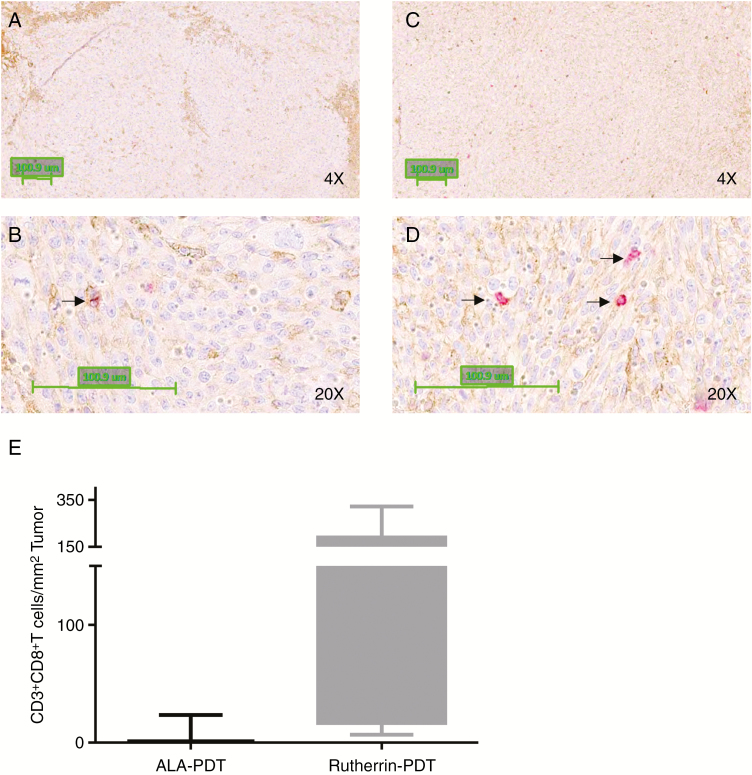
Rutherrin-PDT showed increased intratumor infiltration of CD8^+^ T-cells compared to ALA-PDT. Panel (A) and (B) are the photomicrographs of ALA-PDT-treated tumor sections, and panel (C) and (D) are photomicrographs of Rutherrin-PDT treated tumor sections, stained with anti-CD3 (brown) and Anti-CD8 (red) antibody. Arrows are pointed at tumor border. Panel (E) is the bar graph representing a total number of double positive CD3^+^CD8^+^ T-cells in ALA- and Rutherrin-PDT-treated tumor sections.

A positive correlation between the presence of CD8+ T-cells within the tumor site, including for clinical gliomas,^32^ and improved survival is well documented. An increased number of CD8+ T-cells were noted for Rutherrin-PDT over PpIX-PDT (Figure 5).

### Rutherrin-Mediated PDT Leads to a Significant Increase in Survival

**Fig. 6:**
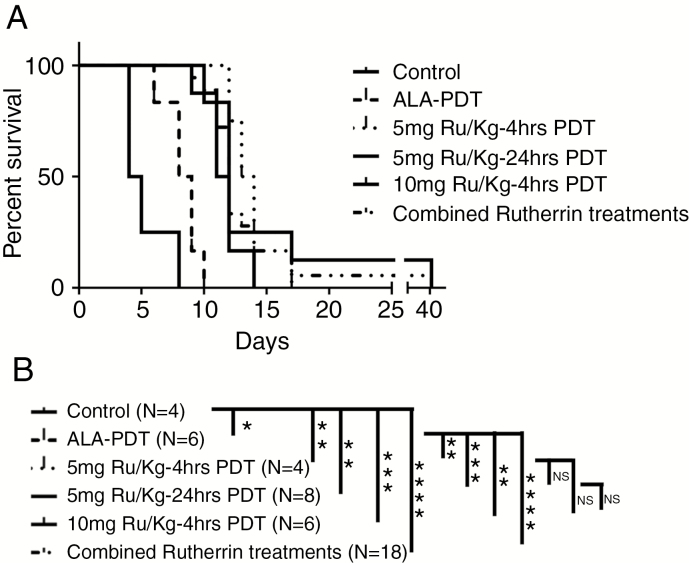
Rutherrin-mediated PDT leads to a significant increase in survival over ALA-mediated PDT. (A) Different cohorts mentioned in the figure were followed up for survival post-PDT treatment. (B) Multiple comparisons of survival curves with the Mantel–Cox Log-Rank test.

At the selected light dose combinations, we did not notice any adverse events. Using a thermocouple placed just beyond the edge of the illumination field, the measured temperature rise during light illumination, was maximal to 39 °C, eliminating light only effect on tissue necrosis. The efficacy of Rutherrin or ALA-induced PpIX PDT at the drug–light interval providing the highest SUR for either photosensitizer according to uptake assays (Figure 3 and Supplementary Table 2)^10^ was determined by following them to a predetermined endpoint. Untreated control rats showed 3–4 mm diameters tumors on 11th day post tumor induction, calculated median survival was 4.5 days (15.5 days post tumor induction). PpIX-PDT-treated rats had significantly improved survival times with a median post-therapy survival of 8.5 days. Whereas, Rutherrin-PDT-treated animals survived a median of 12.5 days. No significant difference was noted between 5 and 10 mg kg^−1^ bw doses and the drug time intervals of 4 or 24 hours for Rutherrin-PDT (Figure 6). Multiple comparisons of different groups are reported in Figure 6B. Photodynamic threshold values of Rutherrin- and ALA-induced PpIX-PDT were calculated as possible causes for survival difference.^22^ The T2 enhancement MRIs post-PDT provided minimum, and maximum enhancement distance from the light source, the average PS concentration with their molar extinction coefficients and PDT irradiation conditions with published optical properties for gray matter^30^ were used in the calculations (Supplementary Table 3). As the entire tumor was necrosed, only the upper boundary threshold values can be provided for Rutherrin at 8.86*10^18^ ± 1.46*10^18^ hv cm^−3^ versus the ALA-Induced PpIX 1.25*10^19^ ± 3.51*10^19^ hv cm^−3^ mediated PDT in the RG2 model. The derived threshold value for normal brain treated with the above Rutherrin-PDT conditions was 3.09*10^18^ ± 2.96*10^17^ hv cm^−3^, whereas that of the ALA-induced PpIX-mediated PDT-treated normal brain was 1.07*10^17^ ± 2.32*10^16^ hv cm^−3^, taking PpIX uptake values as reported by Fisher et al.^10^

## Discussion

The RG-2 LD_50_ determined for ALA-mediated PpIX of 1.54*10^19^ hv cm^−3^ is lower than for the human tongue squamous cell carcinoma,^33^ SCC-4 cells with an LD_50_ = 1.217*10^22^ hv cm^−3^ assuming conversion of all available ALA into PpIX. The calculated RG-2 LD_50_ for Rutherrin mediated with 525 nm light, equal to the ligand-metal energy exchange quantum gap reported by,^34^ is almost three orders of magnitude lower at 2.39*10^16^ hv cm^−3^ again assuming equilibrium between the intracellular and media Rutherrin concentration. Hence, the LD_50_ for ALA-induced PpIX is possibly overestimated, whereas the Rutherrin value may be underestimated. For one the elected growth conditions may be suboptimum for PpIX synthesis,^35^ and more favorable conditions for PpIX accumulation have been shown for other cell lines, and conditions such as lower Young’s modulus in the growth substrate.^35^

Although confining the light delivery to the target by the light attenuation in tissues minimizes morbidity, maximizing the SUR also increases a photosensitizer’s safety.^36^ This holds in particular for central nervous system-based tumors which are invading the surrounding normal eloquent brain areas. Rutherrin is highly selective to RG-2 tumors with a SUR of 20. Considering that each SUR factor 3 provides PDT selectivity over one effective penetration depth a SUR of 20 give close to 2.8 effective penetration depth selectivity or for 808 nm up to 28 mm in gray matter and 15 mm in white matter. In contrast, ALA has a SUR of 10.6 and selectivity just over 2 effective penetration depth at 635 nm equal to 18 mm in gray matter and 7.8 mm. In some of the Rutherrin-injected rats, complete absence to TLD1433 was noted in normal brain, whereas a sufficient PS concentration was still present in the RG-2 tumors (Figure 3, Supplementary Tables 2 and 3).

Light propagation simulations using FullMonte supports the use of 808 nm as the photon density at the tumor boundary is two orders of magnitude higher compared to 635 nm, providing a 10-fold higher photon absorption for Rutherrin-PDT after considering PS concentration and molar extinction coefficient differences. The calculated PpIX-PDT threshold values for normal brain compare well with prior publications.^22^ In addition, the fact that normal brain is more sensitive compared to the tumor model (RG-2) confirms equally prior results whereby the difference is only a factor of 3 for Rutherrin-PDT, much less than for PpIX-PDT permitting a therapeutic selectivity over a larger volume for the former.

The calculated threshold values for the RG-2 tumor versus normal brain are more favorable than those for ALA-induced PpIX as the ratio between tumor, and normal is much smaller for Rutherrin and thus requiring a smaller SUR to achieve selectivity.

Significant differences in the post-PDT T2 MRIs volumes were noted for TLD1433 versus PpIX-mediated therapy. T2 enhancement indicating the extent of edema and non specific inflammation^31^ was double for PpIX-PDT compared to Rutherrin-PDT, pointing to a clinical advantage for Rutherrin-PDT as excessive inflammation of brain tissue can cause post-treatment morbidity, including lethal damage to brain tissue (cerebral death), bleeding and/or stroke due to increased intracranial pressure^31,37–39^ despite patients being given steroids to manage edema. The area of inflammation is also generally associated with a secondary wave of neuronal damage as seen in stroke research and could be further controlled by co-therapies, including hypothermia.^10^

The neuro-oncology consensus is that a high degree of total resection (>98% of the enhancement volume) is required for clinically significant survival improvements.^4^ However, activation of the immune system is essential to target the remaining cancer cells. With potential neoantigens related gene aberrations, combinatorial therapies, including immune modulation, are expected to give better survival over the current standard of care.^40,41^ In clinical cases, it is reported that an elevated level of tumor-infiltrating immune cells, such as anti-cancer CD8+ T-cells, correlates with prolonged survival in GBM patients.^32^

Extracellular calreticulin (CRT) and release of HMGB1 are two important DAMPs required for the induction of immunogenic cell death, responsible for inducing an effective anti-tumor immune response. ALA-PDT-treated glioma did not show a significant increase of extracellular CRT and HMGB1,^42^ whereas, in our unpublished data, we show that Rutherrin-PDT induces a significant increase in extracellular CRT.

Although one report shows improvement of dendritic cell (DC) vaccine-induced T-cell infiltration in tumors, the vaccine was prepared by using ALA-PDT induced apoptotic cells co-culture with DC,^43^ in an in vitro system stimulating DCs, different from the in vivo stimulation in our study. In in vitro produced DCs, one has control over inducing pro-inflammatory DCs and the liberty to use a more stimulatory amount of tumor lysate to co-culture with DCs. Whereas, in in vivo conditions DCs must be recruited to the tumor site in response to PDT mediated cell kill and stimulated to process and present the antigens to T-cells, to induce an effective anti-tumor immune response.

CD8+ T-cells were shown to be essential for protection from tumor challenge in an experimental glioma model vaccinated with DC vaccine.^44^ A high CD8+ T-cell tumor infiltration as observed for Rutherrin-PDT is a possibility of prolonging survival. Supporting our findings of minimal CD8+ T-cell infiltration in ALA-PDT treated tumors, was reviewed previously.^42^

Increased survival in Rutherrin-PDT rats compared to untreated and ALA-induced PpIX-PDT rats, can be partially due to an improved photon density at larger distances from the light source or an increased CD8+ T-cell infiltration in the tumors. Rutherrin-PDT at both TLD1433 doses, 5 and 10 mg kg^−1^ bw of rats, and both drug–light intervals of 4 and 24 hours showed a significant increase in survival over ALA-induced PpIX-PDT. Increased survival with Rutherrin can be partially explained with our in vitro PDT data. ALA-induced PpIX requires three orders of magnitude high absorbed photons to achieve 50% cell kill compared to Rutherrin-PDT. PpIX undergoes rapid photobleaching, whereas TLD1433 is extremely photostable.^15^ There was no significant difference in survival when we used different drug doses and light interventions at 4 and 24 hours post-Rutherrin injection. This observation is clinically advantageous as there is a large drug–light interval window which can accommodate unforeseen surgical circumstances.

In addition, a long period with high SUR in a combination of strong resistance to photobleaching of TLD1433^15^ enables repeated light activation of Rutherrin with a single injection, as supported by the similar SUR and survival time for both Rutherrin doses and drug time intervals (Supplementary Table 2). The observed constant SUR and absolute TLD1433 concentrations were confirmed in a single experiment administering 25 mg kg^−1^ TLD1433 equivalent Rutherrin, suggesting a PS accumulation saturation effect in tissues, potentially due to a cell’s available TfR (Supplementary Figure 2).

Rutherrin is shown to act through both, Type I (oxygen-independent) and Type II (oxygen-dependent) process allowing it to be a powerful and versatile photosensitizer in oxygen-rich or poor environments. Oxygen levels in the normal brain parenchyma are reported to be between 5% and 15%^45^ whereas intratumoral oxygen gradients range from 0.1% in the necrotic tumor center to physiological tissue oxygen concentrations of around 10% at the tumor border. Though the majority of viable and infiltrating GBM cells exist at an oxygen concentration ranging from physiological (10% O_2_) to modest hypoxic (2.5% O_2_).^45^ Hypoxia plays a positive role in the aggression of human glial brain tumors.^45^ To target tumor cells in hypoxic along with cells in normoxic volumes is essential.

Rutherrin-PDT was well tolerated, providing safe and effective treatment of RG-2 glioma. Rutherrin-mediated PDT response appears superior to ALA-PpIX-mediated PDT. In our unpublished clinical study, photosensitizer present in Rutherrin formulation has not shown any phototoxicity, excluding patients from avoiding light sources. With these positive PDT results and the ability to absorb light at longer wavelengths, resistance photobleaching, Rutherrin is a promising photosensitizer formulation to target brain cancer.

## Supplementary Material

vdz006_suppl_Supplimentary_Figure_S1

vdz006_suppl_Supplimentary_Figure_S2

vdz006_suppl_Supplimentary_Information

## References

[1] Brennan CW, Verhaak RG, McKenna A, et al; TCGA Research Network. The somatic genomic landscape of glioblastoma. Cell. 2013;155(2):462–477. 24120142 10.1016/j.cell.2013.09.034 PMC3910500

[2] Stupp R, Hegi ME, Mason WP, et al; European Organisation for Research and Treatment of Cancer Brain Tumour and Radiation Oncology Groups; National Cancer Institute of Canada Clinical Trials Group. Effects of radiotherapy with concomitant and adjuvant temozolomide versus radiotherapy alone on survival in glioblastoma in a randomised phase III study: 5-year analysis of the EORTC-NCIC trial. Lancet Oncol. 2009;10(5):459–466. 19269895 10.1016/S1470-2045(09)70025-7

[3] Huang Z, Cheng L, Guryanova OA, Wu Q, Bao S Cancer stem cells in glioblastoma–molecular signaling and therapeutic targeting. Protein Cell. 2010;1(7):638–655. 21203936 10.1007/s13238-010-0078-y PMC4875273

[4] Stummer W, van den Bent MJ, Westphal M Cytoreductive surgery of glioblastoma as the key to successful adjuvant therapies: new arguments in an old discussion. Acta Neurochir (Wien). 2011;153(6):1211–1218. 21479583 10.1007/s00701-011-1001-x

[5] Stummer W, Stocker S, Wagner S, et al Intraoperative detection of malignant gliomas by 5-aminolevulinic acid-induced porphyrin fluorescence. Neurosurgery. 1998;42(3):518–525; discussion 525. 9526986 10.1097/00006123-199803000-00017

[6] Amelio D, Lorentini S, Schwarz M, Amichetti M Intensity-modulated radiation therapy in newly diagnosed glioblastoma: a systematic review on clinical and technical issues [published correction appears in Radiother Oncol. 2011;99(2):253] Radiother Oncol. 2010;97(3):361–369. 10.1016/j.radonc.2010.08.018 20926149

[7] Fernandes C, Costa A, Osorio L, et al Current standards of care in glioblastoma therapy. In: Vleeschouwer S, eds. Glioblastoma. Chapter11. Brisbane, AU: Codon Publications; 2017. 29251860

[8] Wachowska M, Muchowicz A, Firczuk M, et al Aminolevulinic acid (ALA) as a prodrug in photodynamic therapy of cancer. Molecules. 2011;16(5):4140–4164.

[9] Zeiadeh I, Najjar A, Karaman R Strategies for enhancing the permeation of CNS-active drugs through the blood-brain barrier: a review. Molecules. 2018;23(6):1289–1307. 10.3390/molecules23061289 PMC6100436 29843371

[10] Fisher CJ, Niu C, Foltz W, et al ALA-ppix mediated photodynamic therapy of malignant gliomas augmented by hypothermia. Plos One. 2017;12(7):e0181654. 28759636 10.1371/journal.pone.0181654 PMC5536352

[11] Lim M, Xia Y, Bettegowda C, Weller M Current state of immunotherapy for glioblastoma. Nat Rev Clin Oncol. 2018;15(7):422–442. 29643471 10.1038/s41571-018-0003-5

[12] Carpentier A, Canney M, Vignot A, et al Clinical trial of blood-brain barrier disruption by pulsed ultrasound. Sci Transl Med. 2016;8(343):343re2. 10.1126/scitranslmed.aaf6086 27306666

[13] O’Reilly MA, Chinnery T, Yee ML, et al Preliminary investigation of focused ultrasound-facilitated drug delivery for the treatment of leptomeningeal metastases. Sci Rep. 2018;8(1):9013. 29899537 10.1038/s41598-018-27335-y PMC5998139

[14] Allison RR, Moghissi K Photodynamic therapy (PDT): PDT mechanisms. Clin Endosc. 2013;46(1):24–29. 23422955 10.5946/ce.2013.46.1.24 PMC3572346

[15] Fong J, Kasimova K, Arenas Y, et al A novel class of ruthenium-based photosensitizers effectively kills in vitro cancer cells and in vivo tumors. Photochem Photobiol Sci. 2015;14(11):2014–2023. 25666432 10.1039/c4pp00438h

[16] Kaspler P, Lazic S, Forward S, Arenas Y, Mandel A, Lilge L A ruthenium(ii) based photosensitizer and transferrin complexes enhance photo-physical properties, cell uptake, and photodynamic therapy safety and efficacy. Photochem Photobiol Sci. 2016;15(4):481–495. 26947517 10.1039/c5pp00450k

[17] Dupont C, Vermandel M, Leroy H-A, et al INtraoperative photoDYnamic Therapy for GliOblastomas (INDYGO): study protocol for a phase I clinical trial. Neurosurgery. 2019; 84(6):E414–E419. 30053213 10.1093/neuros/nyy324

[18] Kaufman MB Pharmaceutical approval update. P T. 2017;42(11):673–683. 29089721 PMC5642154

[19] Pichlmeier U, Bink A, Schackert G, Stummer W; ALA Glioma Study Group Resection and survival in glioblastoma multiforme: an RTOG recursive partitioning analysis of ALA study patients. Neuro Oncol. 2008;10(6):1025–1034. 18667747 10.1215/15228517-2008-052 PMC2719000

[20] Fritsch C, Stege H, Saalmann G, Goerz G, Ruzicka T, Krutmann J Green light is effective and less painful than red light in photodynamic therapy of facial solar keratoses. Photodermatol Photoimmunol Photomed. 1997;13(5-6):181–185. 9542754 10.1111/j.1600-0781.1997.tb00226.x

[21] Jacques SL Optical properties of biological tissues: a review. Phys Med Biol. 2013;58(11):R37–R61. 23666068 10.1088/0031-9155/58/11/R37

[22] Lilge L, Wilson BC Photodynamic therapy of intracranial tissues: a preclinical comparative study of four different photosensitizers. J Clin Laser Med Surg. 1998;16(2):81–91. 9663099 10.1089/clm.1998.16.81

[23] Patterson MS, Wilson BC, Graff R In vivo tests of the concept of photodynamic threshold dose in normal rat liver photosensitized by aluminum chlorosulphonated phthalocyanine. Photochem Photobiol. 1990;51(3):343–349. 2356229 10.1111/j.1751-1097.1990.tb01720.x

[24] Schreer A, Tinson C, Sherry JP, Schirmer K Application of alamar blue/5-carboxyfluorescein diacetate acetoxymethyl ester as a noninvasive cell viability assay in primary hepatocytes from rainbow trout. Anal Biochem. 2005;344(1):76–85. 16039980 10.1016/j.ab.2005.06.009

[25] Lilge L, O’Carroll C, Wilson BC A solubilization technique for photosensitizer quantification in ex vivo tissue samples. J Photochem Photobiol B. 1997;39(3):229–235. 9253199 10.1016/s1011-1344(97)00010-9

[26] Cassidy J, Nouri A, Betz V, Lilge L High-performance, robustly verified monte carlo simulation with fullmonte. J Biomed Opt. 2018;23(8):1–11. 10.1117/1.JBO.23.8.085001 30098135

[27] Chang CN, Chen WC, Wei KC, et al High-dose-rate stereotactic brachytherapy for patients with newly diagnosed glioblastoma multiformes. J Neurooncol. 2003;61(1):45–55. 12587795 10.1023/a:1021270201988

[28] Du Le VN, Provias J, Murty N, et al Dual-modality optical biopsy of glioblastomas multiforme with diffuse reflectance and fluorescence: ex vivo retrieval of optical properties. J Biomed Opt. 2017;22(2):27002. 28157245 10.1117/1.JBO.22.2.027002

[29] Strangman GE, Li Z, Zhang Q Depth sensitivity and source-detector separations for near infrared spectroscopy based on the colin27 brain template. Plos One. 2013;8(8):e66319. 23936292 10.1371/journal.pone.0066319 PMC3731322

[30] Yaroslavsky AN, Schulze PC, Yaroslavsky IV, Schober R, Ulrich F, Schwarzmaier HJ Optical properties of selected native and coagulated human brain tissues in vitro in the visible and near infrared spectral range. Phys Med Biol. 2002;47(12):2059–2073. 12118601 10.1088/0031-9155/47/12/305

[31] Mathews MS, Chighvinadze D, Gach HM, Uzal FA, Madsen SJ, Hirschberg H Cerebral edema following photodynamic therapy using endogenous and exogenous photosensitizers in normal brain. Lasers Surg Med. 2011;43(9):892–900. 22006731 10.1002/lsm.21135 PMC4124831

[32] Kmiecik J, Poli A, Brons NH, et al Elevated CD3+ and CD8+ tumor-infiltrating immune cells correlate with prolonged survival in glioblastoma patients despite integrated immunosuppressive mechanisms in the tumor microenvironment and at the systemic level. J Neuroimmunol. 2013;264(1-2):71–83. 24045166 10.1016/j.jneuroim.2013.08.013

[33] Li P, Ke E, Chiang P, Tsai T ALA- or Ce6-PDT induced phenotypic change and suppressed migration in surviving cancer cells. Photochem Photobiol Sci. 2015;10( 1):74–80.

[34] Shi G, Monro S, Hennigar R, et al Ru(II) dyads derived from α-oligothiophenes: a new class of potent and versatile photosensitizers for PDT. Coord Chem Rev. 2015;282( SI): 127–138.

[35] Niu CJ, Fisher C, Scheffler K, et al Polyacrylamide gel substrates that simulate the mechanical stiffness of normal and malignant neuronal tissues increase protoporphyin IX synthesis in glioma cells. J Biomed Opt. 2015;20(9):098002. 26405823 10.1117/1.JBO.20.9.098002

[36] Mehraban N, Freeman HS Developments in PDT sensitizers for increased selectivity and singlet oxygen production. Materials (Basel). 2015;8(7):4421–4456. 28793448 10.3390/ma8074421 PMC5455656

[37] Graham DI, Lawrence AE, Adams JH, Doyle D, McLellan DR Brain damage in non-missile head injury secondary to high intracranial pressure. Neuropathol Appl Neurobiol. 1987;13(3):209–217. 3614546 10.1111/j.1365-2990.1987.tb00184.x

[38] Klatzo I Pathophysiological aspects of brain edema. Acta Neuropathol. 1987;72(3):236–239. 3564903 10.1007/BF00691095

[39] Tweed WA, Overgaard J Cerebral blood flow in patients with intracranial pressure elevation due to traumatic brain edema. Can J Neurol Sci. 1976;3(1):35–37. 1253004 10.1017/s031716710002597x

[40] Hodges TR, Ott M, Xiu J, et al Mutational burden, immune checkpoint expression, and mismatch repair in glioma: implications for immune checkpoint immunotherapy. Neuro Oncol. 2017;19(8):1047–1057. 28371827 10.1093/neuonc/nox026 PMC5570198

[41] Reardon DA, Gilbert MR, Wick W, Liau L Immunotherapy for neuro-oncology: the critical rationale for combinatorial therapy. Neuro Oncol. 2015;17 (Suppl 7):vii32–vii40. 26516225 10.1093/neuonc/nov178 PMC4625894

[42] Hirschberg H, Berg K, Peng Q Photodynamic therapy mediated immune therapy of brain tumors. Neuroimmunol Neuroinflamm. 2018;5:27. 30221185 10.20517/2347-8659.2018.31 PMC6138455

[43] Ji J, Fan Z, Zhou F, et al Improvement of DC vaccine with ALA-PDT induced immunogenic apoptotic cells for skin squamous cell carcinoma. Oncotarget. 2015;6(19):17135–17146. 25915530 10.18632/oncotarget.3529 PMC4627297

[44] Maes W, Rosas GG, Verbinnen B, et al DC vaccination with anti-CD25 treatment leads to long-term immunity against experimental glioma. Neuro Oncol. 2009;11(5):529–542. 19336528 10.1215/15228517-2009-004 PMC2765342

[45] Evans SM, Judy KD, Dunphy I, et al Hypoxia is important in the biology and aggression of human glial brain tumors. Clin Cancer Res. 2004;10(24):8177–8184. 15623592 10.1158/1078-0432.CCR-04-1081

