# Supplementary figures and images for: Efficacy of ruthenium coordination complex–based Rutherrin in a preclinical rat glioblastoma model
Source: Neurooncol Adv. 2019 May 28;1(1):vdz006. doi: 10.1093/noajnl/vdz006 (PMC7212850; doi:10.1093/noajnl/vdz006)

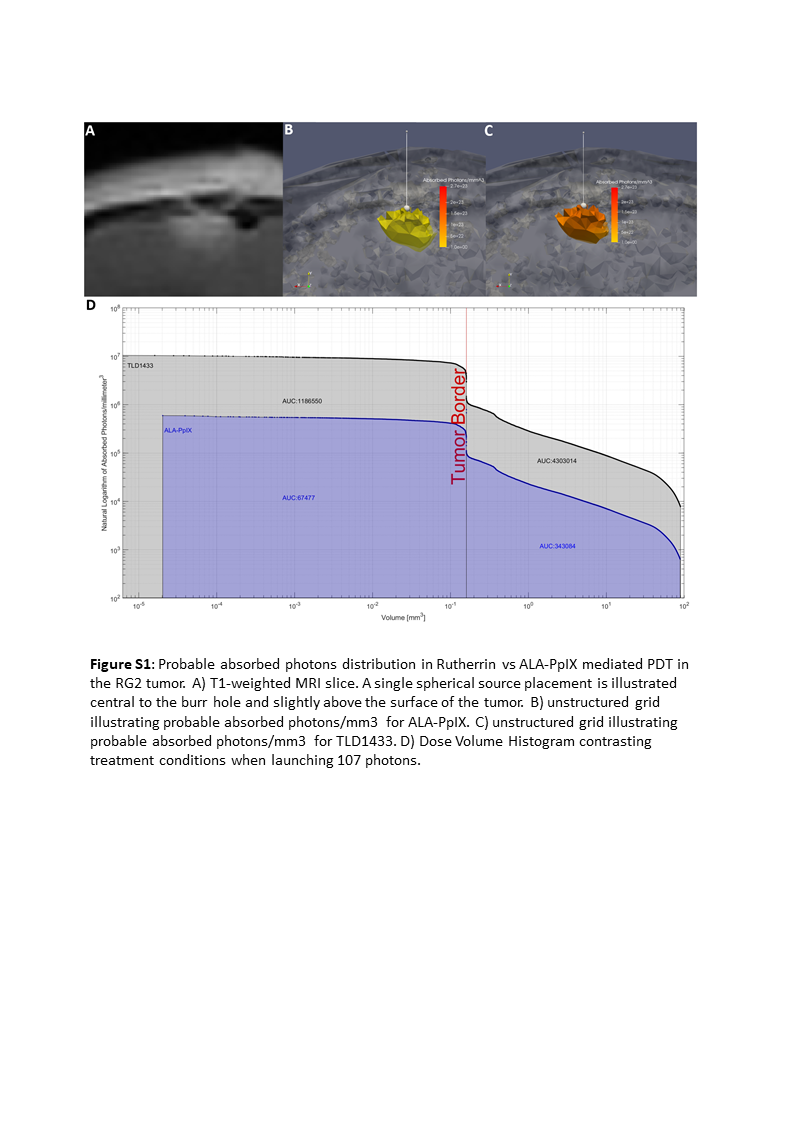

Supplement: vdz006_suppl_Supplimentary_Figure_S1 [file vdz006_suppl_supplimentary_figure_s1.png]

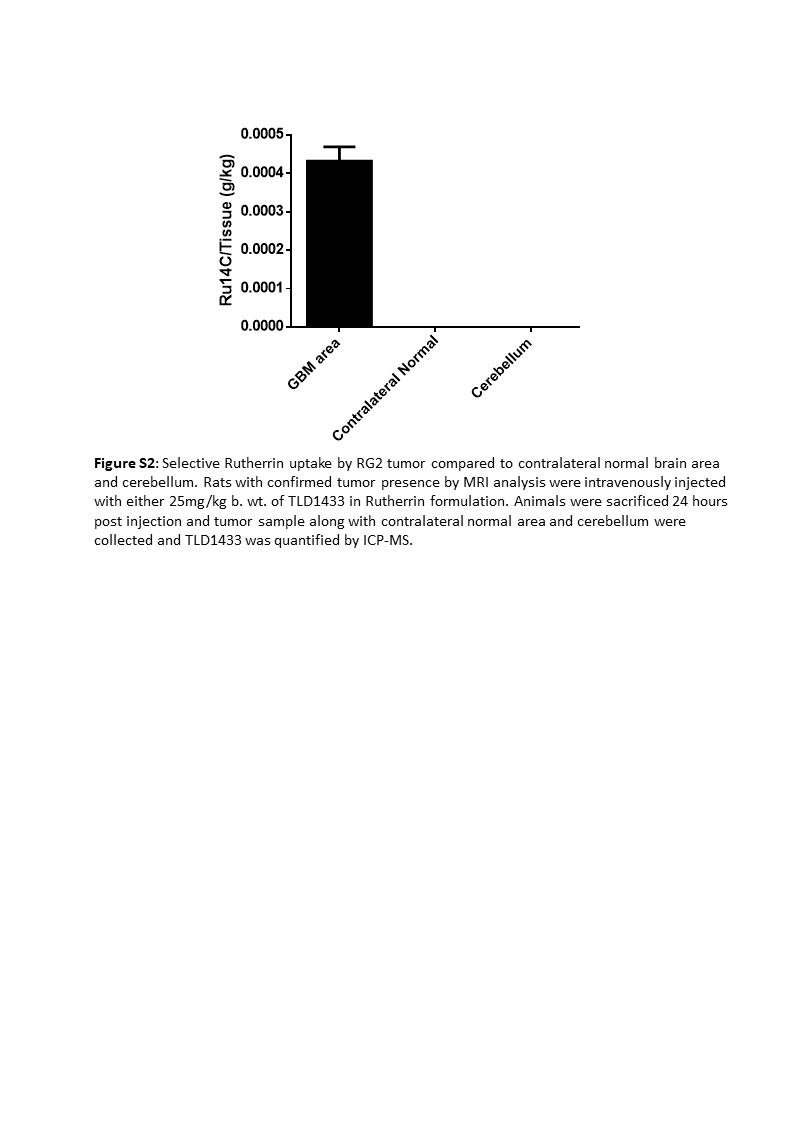

Supplement: vdz006_suppl_Supplimentary_Figure_S2 [file vdz006_suppl_supplimentary_figure_s2.png]
