# Supplementary material for: Efficacy of ruthenium coordination complex–based Rutherrin in a preclinical rat glioblastoma model
Source: Neurooncol Adv. 2019 May 28;1(1):vdz006. doi: 10.1093/noajnl/vdz006 (PMC7212850; doi:10.1093/noajnl/vdz006)
Supplement: vdz006_suppl_Supplimentary_Information [file vdz006_suppl_supplimentary_information.docx]

| **Photophysical properties** | **TLD1433** | **ALA-Induced PpIX** |
| --- | --- | --- |
| **Light absorption** | Through out visible spectrum (380 to 808nm) | Soret Band (405 nm) and Q-Bands (505, 540, 580 and 630 nm) |
| **Singlet oxygen quantum yield** | 0.99 (in acetonitrile) | 0.56 (in PH7.4 PBS with 1% Triton X)^a^ |
| **Fluorescence yield** | Low | High |
| **Maximum Absorption peak** | 416nm | 405 nm |
| **MEC at maximum absorption peak** | 35939 M^-1^cm^-1^ in water | 484 00 M^−1^cm^−1^ in dimethyl formamide^b^ |
| **Solubility in water** | 6mM | 50mg/ml |
| **PH in water** | 5.89 at 0.6mg/ml | 2 at 0.1M solution in water |

1. Journal of Photochemistry and Photobiology B Biology, 1997 ;37(1):131-140
2. European Polymer Journal, 2004 ;40 (10): 2291-2303
3. J Photochem Photobiol B. 1996 ;34(2-3):143-8.

**Table S1**: Photophysical properties of ALA and TLD1433

| **TLD1433 in Rutherrin formulation** | **N** | **RG-2 Tumor**  **(mg-Rutherrin/Kg)** | | | **Contralateral normal brain**  **(mg-Rutherrin/Kg** | | | **Cerebellum**  **(mg-Rutherrin/Kg)** | | |
| --- | --- | --- | --- | --- | --- | --- | --- | --- | --- | --- |
|  |  | **Mean** | **SD** | **Range** | **Mean** | **SD** | **Range** | **Mean** | **SD** | **Range** |
| **4hrs post 5mg TLD1433** | 3 | 3.40 | 1.42 | 1.81 – 0.455 | 0.27 | 0.07.03 | 0.35 - 0.21 | 0.15 | 0.0711 | 0.07 – 0.020 |
| **24hrs post 5mg TLD1433** | 3 | 2.05 | 1.04 | 2.78 – 0.86 | 0.12 | 0.0780 | 0.03 - 0.17 | 0.0775 | 0.0685 | 0 –  0.12 |
| **48hrs post 5mg TLD1433** | 4 | 1.13 | 1.53 | 3.42 - 0.23 | 0.16 | 0.21 | 0.03 - 0.48 | 0.03 | 0.0127 | 0.02 - 0.03 |
|  | | | | | | | | | | |
| **4hrs post 10mg TLD1433** | 3 | 11.64 | 18.42 | 32.91 - 0.93 | 0.51 | 0.53 | 1.08 - 0.02 | 0.57 | 0.55 | 0.16 - 0.36 |
| **24hrs post 10mg TLD1433** | 4 | 0.76 | 0.47 | 1.27 - 0.24 | 0.12 | 0.15 | 7.24E-03 - 0.34 | 0.08 | 5.45E-02 | 0.2 - 0.15 |
| **48hrs post 10mg TLD1433** | 3 | 0.24 | 0.13 | 0.39 - 0.12 | 0.14 | 0.20 | 0 –  0.38 | 0.15 | 6.05E-02 | 0.21 - 0.09 |

**Table S2**: Concentration of TLD1433 in tumor compared to contralateral normal brain and cerebellum

| **Photosensitizer** | **Wavelength (nm)** | **Fluence**  **(J/cm^2^)** | **Concentration (mol/L)** | | **Molar extinction coefficient (m^-1^cm^-1^)** |
| --- | --- | --- | --- | --- | --- |
|  |  |  | Tumor | Brain |  |
| TLD1433 | 808 | 600 | 3.38 E-05 | 4.78 E-06 | 1730 |
| PPIX | 635 | 48 | 6.5 E-07 | 1.3 E-07 | 5121 |

**Table S3:** Concentration of TLD1433 and PPIX in tumor vs normal brain tissue, along with treatment details and molar extinction coefficients of both drugs.

| **Tissue** | **wavelength** | **µa**  **[mm^-1^]** | **µs**  **[mm^-1^]** | **g** | **n** | **µeff**  **[mm^-1^]** |
| --- | --- | --- | --- | --- | --- | --- |
| **GBM** | 635 | 0.04 | 1.95 | 0.85 | 1.39 | 0.20 |
|  | 808 | 0.02 | 1.61 | 0.82 | 1.39 | 0.136 |
| **White matter** | 635 | 0.026 | 9.0 | 0.90 | 1.47 | 0.269 |
|  | 808 | 0.02 | 8.0 | 0.87 | 1.47 | 0.252 |
| **Gray matter** | 635 | 0.018 | 1.90 | 0.88 | 1.36 | 0.115 |
|  | 808 | 0.014 | 1.50 | 0.85 | 1.36 | 0.10 |
| **CSF** | 635 | 0.004 | 0.08 | 0.01 | 1.0 | 0.032 |
|  | 808 | 0.004 | 0.06 | 0.01 | 1.0 | 0.028 |
| **Adipose** | 635 | 0.20 | 25.0 | 0.90 | 1.34 | 1.273 |
|  | 808 | 0.214 | 23.2 | 0.90 | 1.34 | 1.275 |
| **Skull Bone** | 635 | 0.019 | 10.4 | 0.80 | 1.39 | 0.346 |
|  | 808 | 0.012 | 9.20 | 0.80 | 1.39 | 0.258 |

**Table S4**: Tissue optical properties used for light propagation studies.

**Supplementary Methods:**

**SM1. MRI scanning and analysis:**

MR imaging was performed with a 7 Tesla Biospec 70/30 USR system (Bruker Corporation, Ettlingen, DE), equipped with B-GA12 gradient coil insert, 7.2cm inner diameter linearly-polarized cylindrical volume RF transmission coil, and a 4-coil phased array surface receiver coil for RF reception. Following anesthesia induction, rats were positioned prone for imaging, breathing via a nose cone and resting on a 37˚C water bed. Respiration was monitored by pneumatic pillow (SA Instruments, Stony Brook, NY). When required, the tail vein was cannulated by a 27G catheter for injection of 30μl gadolinium- MR contrast (Gd-DTPA, Magnevist, Bayer Corporation, Whippany, NJ, USA) using an injector pump (Ph.D. 2000, Harvard Apparatus, Holliston, MA, USA). T2-weighted imaging was done by Rapid Acquisition with Relaxation Enhancement (RARE) technique with an 85ms echo time (TE), 5200ms repetition time, with a RARE factor of 18, and five averages, requiring ~ 3min imaging time. T1-weighted imaging used the RARE technique with 9.6ms TE, 1000ms TR, a RARE factor of 2, thus 4 averages requiring 4 min 16 sec.
